# Teduglutide for treatment-refractory severe intestinal acute graft-versus-host disease - a multicenter survey

**DOI:** 10.1038/s41409-025-02586-2

**Published:** 2025-04-14

**Authors:** Niklas Brehm, Francesca Biavasco, Johannes Clausen, Johannes Jung, Kristina Maas-Bauer, Ralph Wäsch, Mareike Verbeek, Christoph Nuernbergk, Gabriele Ihorst, Stuart Seropian, Jürgen Finke, Lohith Gowda, Rakefet Sidlik Muskatel, Régis Peffault de Latour, Gérard Socie, Claudia Wehr, David Michonneau, Robert Zeiser

**Affiliations:** 1 Department of Medicine I, Medical Center—University of Freiburg, Faculty of Medicine, University of Freiburg, Freiburg, Germany; 2 Department of Internal Medicine I, Ordensklinikum Linz—Elisabethinen, Linz, Austria; 3 Technical University of Munich, TUM School of Medicine and Health, Department of Medicine III, Hematology and Medical Oncology, Munich, Germany; 4 Clinical Trial Unit, Medical Center—University of Freiburg, Faculty of Medicine, Freiburg, Germany; 5 Yale University School of Medicine, Yale Cancer Center at Smilow Yale New Haven Hospital, New Haven, CT USA; 6 BMT Unit, Hematology-Oncology Division, Schneider Children’s Medical Center of Israel, Petach-Tikva, Tel-Aviv University, Tel-Aviv, Israel; 7 Hôpital Saint-Louis, Hematology and transplantation, Assistance Publique des Hôpitaux de Paris, Paris Cité University, Paris, France; 8 INSERM UMR 976, Human Immunology, Pathophysiology, Immunotherapy, Leukemia institute Paris Saint Louis, IHU Thema2 Paris, Paris, France; 9 INSERM UMR1342, Saint Louis Research Institute, InIdex Immuno-Oncology, SIRIC InSitu, IHU Leukemia Institute Paris Saint Louis, Paris Cité University, Paris, France

**Keywords:** Graft-versus-host disease, Translational research

## Abstract

Intestinal glucocorticoid-refractory (SR) acute (a) graft-versus-host disease (GVHD) causes high non-relapse mortality (NRM) in patients after allogeneic hematopoietic cell transplantation (allo-HCT). Recent preclinical data indicate that acute GVHD causes a loss of intestinal neuroendocrine L-cells leading to reduced levels of glucagon-like peptide-2 (GLP-2). GLP-2 substitution improved GVHD severity and increased Paneth cells and intestinal stem cells in mice. This motivated us to treat patients with refractory intestinal aGHVD using the GLP-2-analogon teduglutide. In this retrospective multicenter survey, 17 patients received teduglutide as salvage-therapy for SR-intestinal aGVHD. The best response (CR or PR) at any time point during and after treatment was 64.7% (11/17) including 41.2% (7/17) CR and 23.5% (4/17) PR. At a median follow-up of 28 weeks after teduglutide 10/17 patients are alive. Most patients experienced an increase of the albumin serum level within 2 months after the first teduglutide dose, including patients who clinically did not respond to teduglutide treatment. No specific teduglutide-related toxicity was observed. Our retrospective analysis suggests that teduglutide is safe and has activity in a fraction of patients with intestinal SR-aGVHD, which needs validation in a prospective trial.

## Introduction

Despite the standard prophylaxis including a combination of a calcineurin inhibitor, an antimetabolite (MMF/MTX) and T cell depletion (ATG or PTCy) 30 to 60% of patients undergoing allogeneic haematopoietic cell transplantation (allo-HCT) develop acute graft-versus-host disease (aGVHD) [1–3]. The three target organs of aGvHD are the skin, the intestine and the liver. The cumulative incidence of gastrointestinal aGVHD was reported to be around 60% [4]. Intestinal SR-aGVHD has the highest mortality compared to skin and liver aGVHD and significantly contributes to post-transplant-mortality and morbidity [1, 5–7]. Apart from the risk of malignancy relapse, aGVHD still remains a major cause of death after allo-HCT [5, 8]. Initial therapy of aGVHD is still the use of high-dose systemic glucocorticoids (GC) [9–11]. 35–60% of aGVHD patients will achieve a durable response to corticosteroids [3, 12]. Patients who do not achieve an adequate response to the first-line GC therapy have an increased risk of mortality and therefore a need for other treatment options [10].

Based on a randomized phase III trial the Janus kinase 1 and 2 inhibitor ruxolitinib was approved for SR-aGVHD [8, 13]. If the patient is refractory to both GC and ruxolitinib the treatment differs in various centers [9, 14, 15]. Many of these therapies are potent immunosuppressive agents and may cause high infection rates and other severe side effects [8, 10]. Based on a better understanding of the pathogenesis of aGVHD, strategies other than immunosuppression are being explored, especially approaches that aim to restore tissue homeostasis and promote the healing of aGVHD-related tissue damage [16].

Glucagon-like peptide (GLP-2) is an enteroendocrine hormone, produced and secreted by L-cells mainly in the distal ileum and proximal colon [17, 18]. It has been shown that GLP-2 has a tissue protective and regenerative function by promoting the blood flow in the mesenteric vessels, causing a higher crypt depth, higher villi and promoting mucosal growth [19, 20]. Lower GLP-2-levels were observed in mice developing GVHD and low numbers of L cells in intestinal biopsies and high serum levels of GLP-2 were connected to increased nonrelapse mortality in allo-HCT patients [18]. GLP-2 substitution caused microbiome changes, enhanced the production of antimicrobial peptides and stimulated the regeneration of Paneth cells and intestinal stem cells in mice [18]. Additionally, GLP-2 reduced the expression of apoptosis-related genes and expanded intestinal organoids [18].

Teduglutide is a GLP-2 analog which is already approved in Europe, Japan and the US for use in pediatric short bowel syndrome [21]. In multiple mouse models teduglutide showed reduced de novo aGVHD and steroid-refractory GVHD without compromising the graft-versus-leukemia effect [18]. Also the SR-GVHD-related death and intestinal GVHD histopathology in the mouse model was reduced [18]. A recently reported series including 3 pediatric patients showed that teduglutide treatment was connected to a major improvement of their intestinal GVHD on the clinical and histological level [17]. The prospective STARGAZE trial (ClinicalTrials.gov ID: NCT05415410) is analyzing the effect of the GLP-2 analog apraglutide to treat severe intestinal aGVHD, when combined with ruxolitinib for SR-aGVHD affecting the intestinal tract.

Here we performed a multicentre survey on the use of teduglutide in patients with severe intestinal SR-aGVHD that had failed several lines of treatment. We found that teduglutide has activity in a main fraction of patients with SR-aGVHD who were refractory to ruxolitinib and other therapeutical approaches without any new signs of toxicity.

## Methods

### Ethics approval and consent to participate’ statement

All methods were performed in accordance with the relevant guidelines and regulations. Approval has been obtained from the ethics committee of Freiburg University Medical Center (24-1470-S1). Informed consent was obtained from all participants.

### Analysis approach

We retrospectively analyzed the GvHD-Data-Base of Freiburg University Medical Center and other Centers for intestinal GvHD patients who received teduglutide treatment. Patients from the different transplant centers included Freiburg (*n* = 5), Linz (*n* = 1), Munich (*n* = 1), Saint Louis Paris (*n* = 9) and Petach-Tikva (*n* = 1). All data were anonymized. The use of teduglutide in the treatment of intestinal aGvHD was off-label and used upon failure of standard treatment (Table 1). The patients received daily teduglutide of 0.05 mg/kg subcutaneously. The duration of treatment varied in between 5 and 81 days and the decision when to stop teduglutide was made by the attending physician based on response status. Ileocolonoscopy-biopsy was performed in one of the patients treated in Freiburg before and during teduglutide-treatment. The indications for the intervention were GVHD diagnosis and GVHD response, respectively.

**Table 1:** Patient characteristics and GVHD severity.

Number of patients	17
Variable	
Patient age (years)	Median (range)
	54 (2–72)
Gender	absolute number (%)
Female	8 (47.1)
Male	9 (52.9)
aGVHD grade before teduglutide treatment	Median (range)
	III (III-IV)
aGVHD gut staging before teduglutide treatment	Median (range)
	4 (2–4)
Number of previous GVHD treatments	Median (range)
	5 (2–9)
Types of GvHD-treatment	absolute number (%)
Corticosteroids	17 (100.0)
Ruxolitinib	15 (88.2)
TNF-Inhibitors (Infliximab, Etanercept)	10 (58.8)
α1-antitrypsin	9 (52.9)
ECP	8 (47.1)
Vedolizumab	6 (35.3)
Other Immunosupressions (CSA, Everolimus, Tacrolimus, MMF, MTX)	7 (41.2)
Inolimumab	2 (11.8)
Microbial transfer	2 (11.8)
MSCs	2 (11.8)
ATG	1 (5.9)
Abatacept	1 (5.9)
Organs involved	Median (range)
	2 (1–3)
Organ affection	absolute number (%)
Gastrointestinal tract	17 (100.0)
Skin	9 (53.0)
Liver	2 (11.8)
**Underlying disease**	absolute number (%)
Myelodysplastic syndrome	7 (41.2)
Acute myeloid leukemia	6 (35.3)
Lymphoma	2 (11.8)
Acute lymphoblastic leukemia	1 (5.9)
Juvenile myelomonocytic leukemia	1 (5.9)

Patients demographics, GvHD grading, treatment regime, response, albumin level, follow- up, were collected and data was analyzed using GraphPadPrism (10.02.3). The error bars in the graphs indicate the SEM (standard error of the mean). Overall survival analysis was calculated as time from start of treatment with teduglutide until death from any cause or last documented visit (censored observations) and was estimated using the Kaplan Meier method.

## Results

We retrospectively analyzed patients with severe steroid-refractory intestinal aGvHD who received treatment with the GLP-2 analog teduglutide from and different transplantation centers. In total the data of 17 patients were analyzed including 9 males and 8 females with a median age of 54 years (Table 1). The most common underlying diseases were MDS (41.2%) and AML (35.3%). Most of the patients had a severe intestinal GvHD with a median GVHD grading of III and an intestinal aGVHD staging of 4 ranging between 2 and 4 (Table 1). The patients had received 2–9 treatments before teduglutide was started. All patients had steroid-refractory acute GVHD and apart from one individual treated before EMA approval of ruxolitinib and the two-year-old child, all patients had received ruxolitinib as a second-line therapy. Prior to teduglutide multiple other treatments were used (median 5 different treatments/patient). Prior treatment included TNF-inhibitors (infliximab or etanercept, 10/17), α1-antitrypsin (9/17), extracorporeal photopheresis (8/17), raising immunosuppression dose or changing to another substance (7/17) or vedolizumab (6/17) (Table 1). Other regimen including microbial transfer, ATG or mesenchymal stroma cells were also applied (Table 1).

**Fig. 1:**
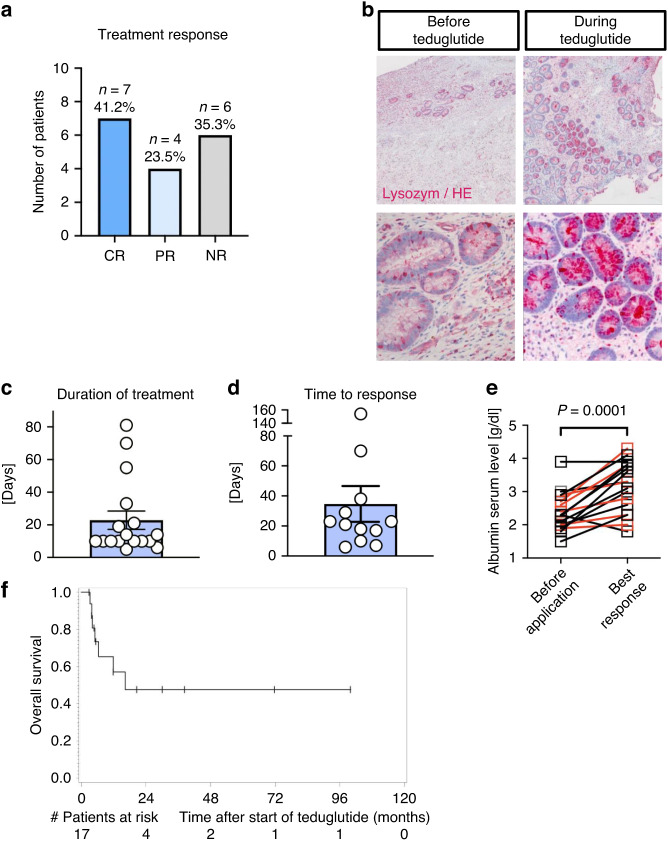
Teduglutide in patients with intestinal SR-aGVHD. **a** Bar graph showing the clinical or histological response after teduglutide treatment. CR complete response, PR partial response, NR non-responder. n indicates the number of patients per group. % indicates the relative number of patients per group. **b** Representative ileocolonoscopic-samples pre- and during teduglutide-treatment. **c** Bar graph showing the duration of treatment in days. Each dot represents one patient. The error bars indicate mean with SEM. **d** Bar graph indicating the time to response in days. Each dot represents one patient. The error bar indicates mean with SEM. **e** Graph describing the serum albumin levels in g/dl before teduglutide application and the best response within two months after first application. The connected dots indicate the two timepoints of one patient. The red dots indicate patients that were non-responders. A paired t-test was performed. *P* = 0.0001. **f** Kaplan Meier estimation of overall survival from start of teduglutide treatment.

Teduglutide was added to the current treatment that had not shown a response. Responses were seen in 64.7% of the patients (11/17) including a clinical or a histological response or both (Fig. 1a). 7/17 patients achieved a complete remission (CR) while 4 individuals achieved a partial response (PR) (Fig. 1a). The partial response was defined as a histological improvement of the histological grading or as a clinical improvement with at least one stage less than before teduglutide treatment. An ileum biopsy was performed before and during teduglutide treatment in one patient but not in all patients to reduce the risk for perforation of the highly inflamed intestines. The pre-treatment biopsy showed signs of tissue injury indicative for a severe aGVHD including cryptitis, ulcerations, epithelial apoptosis and a low number of Paneth cells (Fig. 1b). Conversely, the sample collected during teduglutide treatment showed less signs of aGVHD and a rising number of Paneth cells (Fig. 1b).

Teduglutide was applied in the standard dose for short-bowel syndrome which is 0.05 mg/kg through subcutaneous injection once a day. The duration of treatment varied between 5 and 81 days with a mean of 22.8 days of treatment and a median of 10 days (Fig. 1c). Most of the patients showed a response within the first 40 days (Fig. 1d). Two patients achieved response after 70 and 154 days, respectively.

Albumin is an indicator for intestinal absorption and a prognostic biomarker for patients developing aGVHD [22]. We observed rising albumin levels in intestinal GVHD patients when comparing the serum concentration before teduglutide-application with the best response within the first two months both in responders (marked in black) and in four of the six non-responders (marked in red) (Fig. 1e). Figure 1f shows estimated OS rates after one year of 57.1% (95% confidence interval, CI 27.7–78.3%), after two years 47.6% (95% CI 19.5–71.4%). At the time of data cut-off 10 out of 17 patients are still alive. The longest follow-up had a patient who was still living more than 96 months after teduglutide-application.

Regarding the adverse events during treatment, there was just one patient with CMV-reactivation already before teduglutide application that required treatment before and during treatment. The other 4 patients with CMV-reactivation were not requiring any treatment (Table 2A). There was also no treatment-associated cytopenia. Teduglutide treatment of one additional patient was discontinued after the first dose due to adverse events including headache and constipation (Table 2B). Even after re-exposure the treatment had to be terminated again due to constipation and headache. The teduglutide-treatment of the other 17 patient was well tolerated, and no major adverse events were reported in this cohort.

**Table 2:** A. Adverse events occurring during treatment with teduglutide. B. Characteristics of the patient developing AEs when treated with teduglutide.

A	
AEs	
CMV reactivation (no treatment necessary)	absolute number (%) 4 (23.5)
Relapse of Malignancy (during/after treatment)	absolute number (%) 3 (17.6)
**B**	
**Patient characteristics**	
Age	32
Gender	Male
Underlying disease	High risk MDS
aGVHD grade before teduglutide treatment	III (lower GI tract GVHD)
Previous GvHD treatments	Corticosteroids
	Vendolizumab
	Ruxolitinib
	ECP
	Microbial transfer
	Etancercept/Infliximab
AEs	headache
	constipation

## Discussion

Intestinal aGVHD remains a life-threatening complication after allo-HCT, especially if refractory to glucocorticoid therapy, associated with high morbidity and poor prognosis. Looking for new approaches, especially targeting the gastrointestinal homeostasis, we analyzed retrospectively patients with severe intestinal SR-aGvHD who received treatment with the GLP-2 analog teduglutide from different transplantation centers. The intestinal tract is a key site for immune dysregulation during aGVHD involving the release of damage associated patterns (DAMPs) like ATP [23] and uric acid [24] and an infiltration by innate immune cells including neutrophils [25], monocytes and macrophages [26]. Neutrophils may damage intestinal cells but also contribute to donor T-cell priming [27] and a fraction of neutrophils has shown protective activity against aGVHD [27].

Within our study cohort of 17 heavily pretreated patients with severe intestinal aGVHD, we could observe an overall response rate of 64.7% (11/17), including 7 CRs (7/17). This effect might be due to intestinal epithelial regeneration without additional immunosuppression. These findings provide a therapeutic rationale for GLP-2 analogs as a tissue regeneration approach in the treatment of aGVHD [18]. On the other hand, more than a third of the cohort did not show any response to teduglutide which could be due to the often relatively short treatment time. Out of the 6 non-responders 5 were treated with the shortest teduglutide treatment (2 weeks or less), which might have influenced the missing response. Therefore, the ideal treatment duration with teduglutide still must be defined and it is possible that an earlier treatment will increase the response rates.

Our study provides a median follow-up of 28 weeks after teduglutide application. This follow-up is long enough to evaluate the response to the teduglutide treatment. The individual follow-up of one patient was 6 years and still has no relapse or colon cancer at this follow-up time.

Comparable to the so far reported three pediatric patients our study did not find any adverse events relatable to teduglutide except for the additional patient described below, indicating that it was well tolerated. It is described that teduglutide increases the splanchnic blood flow and therefore could theoretically increase the GVHD associated lower GI-bleeding [17], which we did not observe. As already described above one additional patient is reported who did not tolerate the treatment after two attempts due to headache and constipation.

Prior studies on patients with short bowel syndrome receiving teduglutide did not show an increased risk for colon cancer [28]. Nevertheless a study of 35 short bowel patients indicates that teduglutide might increase the risk of developing polypoid intestinal lesions in patients after a mean treatment duration of 23 months [29]. In contrast teduglutide treatment in our cohort for aGVHD was not longer than 2 months.

The safety and efficacy of the GLP-2 analog apraglutide for steroid-refractory lower intestinal tract aGVHD was analyzed in the prospective STARGAZE trial (ClinicalTrials.gov ID: NCT05415410). The interim analysis of this trial reported that over 50% of the patients treated with apraglutide in combination with ruxolitinib had a clinical response on day 28 and that responses were maintained on days 56 and 91 [30] supporting the concept of combining GLP-2 analogs with ruxolitinib. Apraglutide has a particularly low clearance and therefore prolonged elimination half-life in comparison with teduglutide in different mammals. In vitro, apraglutide was 2-fold more potent for activation of the hGLP-2 receptor compared with teduglutide [31]. In healthy volunteers apraglutide also showed slower absorption, reduced clearance and higher protein binding in comparison to native GLP-2 [32]. These aspects might be beneficial for patients enabling a reduced dosing frequency [31]. The safety of apraglutide and teduglutide analyzed in a meta-analysis were comparable, as both drugs caused only mild to moderate AEs with no statistical significance regarding the incidence in comparison to the control-group [28]. For teduglutide the most commonly reported adverse events in patients with short bowel syndrome were abdominal discomfort, headache, nausea, vomiting and pyrexia while the most common adverse events with apraglutide were polyuria and GIT stoma related complications [28]. The STARGAZE trial showed similar safety results for apraglutide in GvHD patients with an acceptable safety profile with no serious events caused by apraglutide [30].

A recent analysis showed that ruxolitinib resulted in a lower nonrelapse mortality (NRM) than non-ruxolitinib therapies [33]. In this analysis patients with high MAGIC algorithm probabilities (MAPs) experienced high nonrelapse mortality regardless of ruxolitinib-treatment [33]. The majority of our patients (88.2%) received ruxolitinib as a second-line treatment. In future studies one could apply the MAGIC algorithm to our patients to clarify whether patients with high MAPs benefit from teduglutide more than from other treatments and to evaluate whether the MAGIC algorithm could predict teduglutide-response in patients in the future.

In none of the patients who responded after teduglutide application ruxolitinib or other GvHD therapies were started shortly before or during teduglutide application.

The limitation of our analysis includes the retrospective design, the small sample size of 17 patients and the variation in the duration of teduglutide treatment. However, our analysis provides the so far largest real-world data report on the use of an FDA approved GLP-2 analog in patients with intestinal aGVHD having failed multiple therapies, including its relative safety when used over prolonged duration. It is currently unclear how long teduglutide should be administered. Our data suggest that some patients may already benefit from a 2 to 3 week treatment period. In two patients the time to response was significant longer than the duration of treatment. We cannot clarify in these two patients if the response was possibly due to other later treatments.

We conclude that teduglutide is a promising therapy for patients with intestinal SR-aGVHD not responding to multiple agents including ruxolitinib. Teduglutide is non-immune suppressive and apart from one patient with headache and constipation it did not cause major side effects in the patient cohort that we report here. Future trials have to clarify if GLP-2 analogs may be helpful in earlier lines for intestinal aGVHD therapy or even as prophylactic therapy in patients with a high GVHD risk. Our findings offer impetus and novel avenues to further test mechanistically driven minimally immunosuppressive agents like teduglutide to be combined with other tolerance enhancing drugs to mitigate NRM and morbidity in alloHCT recipients an enhance its further application.

## Data Availability

All data generated or analysed during this study are included in this published article.
